# Medicinal Plants, Phytochemicals, and Their Impacts on the Maturation of the Gastrointestinal Tract

**DOI:** 10.3389/fphys.2021.684464

**Published:** 2021-07-30

**Authors:** Nyasha Charity Mukonowenzou, Kehinde Ahmad Adeshina, Janine Donaldson, Kasimu Ghandi Ibrahim, Dawoud Usman, Kennedy Honey Erlwanger

**Affiliations:** ^1^Department of Anatomy and Physiology, National University of Science and Technology, Bulawayo, Zimbabwe; ^2^Department of Physiology, Faculty of Basic Medical Sciences, College of Health Sciences, Usmanu Danfodiyo University, Sokoto, Sokoto, Nigeria; ^3^Centre for Advanced Medical Research and Training, Usmanu Danfodiyo University, Sokoto, Sokoto, Nigeria; ^4^Faculty of Health Sciences, School of Physiology, University of the Witwatersrand, Johannesburg, Johannesburg, South Africa

**Keywords:** development, gastrointestinal tract, gut microbiota, immunity, maturation, phytochemicals

## Abstract

The gastrointestinal tract (GIT) is the first point of contact for ingested substances and thus represents a direct interface with the external environment. Apart from food processing, this interface plays a significant role in immunity and contributes to the wellbeing of individuals through the brain-gut-microbiota axis. The transition of life from the *in utero* environment, to suckling and subsequent weaning has to be matched by phased development and maturation of the GIT; from an amniotic fluid occupancy during gestation, to the milk in the suckling state and ultimately solid food ingestion at weaning. This phased maturation of the GIT can be affected by intrinsic and extrinsic factors, including diet. Despite the increasing dietary inclusion of medicinal plants and phytochemicals for health benefits, a dearth of studies addresses their impact on gut maturation. In this review we focus on some recent findings mainly on the positive impact of medicinal plants and phytochemicals in inducing precocious maturation of the GIT, not only in humans but in pertinent animals. We also discuss Paneth cells as mediators and potential markers of GIT maturation.

## Introduction

The gastrointestinal tract (GIT), functions in the digestion of food, absorption of nutrients, and excretion of waste products (Liao et al., 2009). The role of the GIT with regards to the maintenance of overall health and well-being, however, extends far beyond its digestive and absorptive capacity. The human GIT is colonized by up to 100 trillion microorganisms (Bresalier and Chapkin, 2020), including bacteria, fungi, viruses, protists and archaea (Quigley, 2017). The gut microbiota are involved in the regulation of host energy and metabolism, the development and maintenance of host immune function, as well as the synthesis of nutrients and essential vitamins (Wallace, 2020), which in turn influence normal host physiology.

Functional gut maturation is characterized by morphological and biochemical changes in the gut during the early postnatal life. Such changes are classically triggered by hormones or nutrition (Sangild, 2001). Enteral nutrition, ranging from colostrum, natural milk, formulas, elemental diets, supplements, and phytochemicals, exert selective effects on gut maturation to a degree dependent on the cue itself (Sangild, 2001; Celi et al., 2017). Notably, the small intestinal crypts bear Paneth cells that have been shown to play key roles in GIT function (Chung and Raffatellu, 2019). These cells contribute to the integrity and cellular activities of the small intestinal crypts (Srugo et al., 2019) through secretion of growth factors (Spatz and Mills, 2019). Additionally, they regulate the intestinal microflora and have chemosensory functions (Vaishnava et al., 2008; Roura et al., 2019) and influence gut cell differentiation and maturation (Sato et al., 2011; Mei et al., 2020).

The gut microbiota are important in the establishment and regulation of non-enteral and enteral functions specifically with regards to tolerance of the host to food and other orally ingested antigens and the promotion of tissue repair through Toll-like receptor activation (Rakoff-Nahoum et al., 2004; Belkaid and Hand, 2014). The gut microbial community is first established at birth and continues to develop as the gut matures. The suckling period is characterized by a low microbiota biodiversity and the milk-based diet favors the dominance of *Bifidobacteria* and *Lactobacilli* (Cresci and Bawden, 2015). At weaning, the gut microbiota “matures” somewhat and there is an increase in gram-negative bacteria following the transition to solid food and an increase in overall species diversity, dominated by *Firmicutes* and *Bacteroidetes* (Ottman et al., 2012; Jain and Walker, 2015).

The “far-reaching” effects of perturbations in gut bacteria composition can be mediated via the brain-gut-microbiota axis. This axis is a bidirectional communication between the central nervous system and the enteric nervous system (Quigley, 2018; Tait and Sayuk, 2021). Pathways involved in this communication include the vagus nerve, and through chemical messengers such as peptides, hormones, and neurotransmitters, produced in both gut and brain (Cani and Knauf, 2016; Wallace, 2020). Changes in gut microbiota composition can affect the secretion of these gut-derived chemical messengers, affecting communication between the gut and the brain, hence, brain-gut-microbiota axis (Wallace, 2020). Endogenous and exogenous factors affecting the composition of the gut microbiota include diet, local pH and oxygen supply, immune responses, host genetics, stress, and other environmental factors (Hasan and Yang, 2019).

The gut maturation process is different for precocial and altricial species. Precocial species, including pigs and humans, have relatively long gestational periods and are born more “mature” in terms of their gut compared to altricial species, such as rodents, which have a much shorter gestational period and undergo extensive postnatal development (Pacha, 2000; Baintner, 2007). Although ontogenic patterns of GIT maturation bear similarities across species, some variability in timing exists.

Notably, the intestine undergoes most of the functional and structural changes in line with the changes in diet that occur after birth or upon weaning. Adaptations to enteral feeding during the suckling period include major changes in gastrointestinal structure, motility, digestive and absorptive function (Aynsley-Green, 1989). Also, enteral feeding promotes normal gut digestive enzyme activity, much earlier than in premature infants (Green and Nasset, 1980). The milk suckled during the suckling period contains hormones and growth factors which enhance gut function to adapt to the enteral feeding (Lucas and Mitchell, 1980). Upon weaning, the GIT modifies to adaptation for the digestion of a “solid” (adult) diet (Prykhodko et al., 2015). Structural and functional changes which occur within the GIT include: the replacement of fetal-type vacuolated enterocytes with adult-type non-vacuolated enterocytes in the distal small intestine; a change in intestinal brush-border disaccharidases from lactase to sucrase and maltase; and the replacement of immature, neonatal-Fc-receptor (FcRn) expressing enterocytes for mature enterocytes with low FcRn expression in the proximal small intestine, resulting in “gut closure” (Prykhodko et al., 2015; Sureda et al., 2016).

Diet is a primary factor which regulates the composition of gut microbiota and affects the gut maturation process. Currently, medicinal plants and their derived phytochemicals have gained popularity in most diets as prophylactic agents or treatments for several disorders. As such, the interrelationship between phytochemicals, gut microbiota and the gut maturation process needs further exploration.

## Impact of Medicinal Plants and Phytochemicals on the Development and Maturation of the Gastrointestinal Tract

The natural process of gut maturation can be induced precociously by abrupt weaning (Lee and Lebenthal, 1983), corticosteroids (Martín et al., 1993), provocation with pancreatic or pancreatic-like proteases (Prykhodko et al., 2015; Sureda et al., 2018) and polyamines (Dufour et al., 1988; Bekebrede et al., 2020), amongst other factors. Additionally, several studies indicate that a diverse range of medicinal plants and phytochemicals have potential as maturational agents to the developing gut (Supplementary Table 1). Oral administration of an aqueous calyx extract of *Hibiscus sabdariffa* to suckling rats for 9 days, resulted in early maturation of the small intestine and the cecum, as demonstrated by significantly increased organ weights relative to the control group (Ibrahim et al., 2017). Dangarembizi et al. (2014) demonstrated a dose-dependent trophic effect on cecal mucosal layers of Sprague Dawley pups following the oral administration of a methanolic extract of *Ficus thonningii* for 7 days, starting from postnatal day 6. The findings were attributed to phytochemicals that can stimulate the release of regulatory peptides which then favor the proliferation of parietal cells, thus accelerating the growth of the gut mucosa (Jain and Samuelson, 2006). Similarly, both aqueous and ethanolic extracts of *Aloe vera*, administered during the suckling period for 8 days, accelerated the growth of the cecum and its mucosal layers in rats (Beyaa et al., 2012). Increased organ weight, changes in crypt length, surface area and diameter of the intestines are ontogenic changes that occur naturally at weaning or during the postweaning period (St Clair and Osborne, 1985; Goodlad and Wright, 1990). Early appearance of these features is indicative of precocious maturation. *Aloe vera* (Kar and Bera, 2018) and *Ficus thonningii* (Ahur et al., 2010) contain polysaccharides which may undergo gut microbial fermentation to yield short chain fatty acids (SCFA) (Steed and Macfarlane, 2009; Feng et al., 2018) especially butyrate, which has trophic effects on the cecal mucosa (Lobo et al., 2007). Similarly, neonatal rats orally administered an ethanolic extract of African potato (*Hypoxis hemerocallidea*) developed heavier ceca compared to their control counterparts (Erlwanger and Cooper, 2008). It is important to note though that in the studies cited, maturation markers were not investigated. However, the fact that the extracts did induce some degree of maturational changes in the gut is pertinent.

Phytochemicals such as phytohemagglutinin (PHA), seaweed-derived polysaccharide and allicin have also been used to induce precocious gut maturation (Supplementary Table 1). PHA induces precocious maturation of the gut by promoting growth of the small intestine (Linderoth et al., 2005; Sureda et al., 2018). Its administration to neonatal rats stimulates increased crypt number (Linderoth et al., 2005) and crypt depth (Linderoth et al., 2006; Prykhod’ko et al., 2009). Enteral PHA exerts a time dependent effect on the intestinal function of neonatal rats Linderoth et al. (2006). The early effects, within 24 h, PHA blocks both the receptor-mediated endocytosis and the unspecific absorptive capacity of the intestinal epithelium by binding to the enterocytes (Linderoth et al., 2006; Sureda et al., 2018). For the late effects, after 72 h, there is absence or reduced binding of PHA to the enterocytes (Linderoth et al., 2006), and a decreased absorptive endocytosis capacity resulting from the transition of the fetal-type enterocytes to the adult-type, corresponding to gut closure (maturation) (Linderoth et al., 2005, 2006; Prykhod’ko et al., 2009). The reduced absorptive capacity reflects an accelerated growth of the intestine since the uptake of IgG is mediated by fetal-type enterocytes expressing FcRn receptors and not the adult type which lacks the receptor (Sureda et al., 2016).

Maturation of the gut is characterized by increased digestive capacity at weaning with resultant changes in the properties of brush border enzymes and pancreatic enzyme secretion (Henning, 1981; Rakhimov et al., 2002). PHA administration stimulated increased trypsin (Sureda et al., 2018), maltase and sucrase activities (Prykhod’ko et al., 2009) and an adult-like disaccharidase pattern (Linderoth et al., 2005) in suckling rats, prior to weaning. Similarly, the number of proteins increased in the brush-border membranes of the intestine in neonatal rats receiving PHA during the pre-weaning period (Kruszewska et al., 2003). Neonatal administration of allicin (from garlic *Allium sativum*) to suckling piglets for 6 days, beginning on the second day of birth, resulted in precocious development of the small intestine (Tatara et al., 2008). Supplementary Table 1 provides a summary of some studies which investigated the role of medicinal plants in precocious maturation of the gastrointestinal tract.

## Potential Benefits of Precociously Induced GIT Maturation

The use of stimulants of precocious gut maturation in early nutrition can be advantageous, particularly to preterm infants (Sangild et al., 2013). Nutrition has also been identified as a means of boosting immunity, ameliorating weaning stress, preventing the entry of noxious agents into the GIT and preventing the development of Necrotizing Enterocolitis (NEC) and postweaning diarrhea, through the promotion of gut maturation (Sangild, 2001; Claud, 2009; Szajewska and van Goudoever, 2014; Wopereis et al., 2014; Celi et al., 2017; Kenyon and Cranston, 2017).

### Boosting Immunity

The development of the body’s immunity largely coincides with the development of the GIT as it houses 70–80% of gut associated lymphoid tissues (GALT). This immune organ development (both innate and adaptive) is incomplete in early life, even amongst precocial humans (Scholtens et al., 2012). As with preterm infants, prolonged gut underdevelopment translates to reduced resilience against food borne pathogens. Exposure to certain nutritional cues impacts the immune system and intestinal surface barrier to functionally develop as a frontline defense against harmful pathogens, while tolerating commensals. Over time, this is made possible by the presence of micro-environmental factors from the gut microbiota and food (Martin et al., 2010; Wopereis et al., 2014). Consuming milk and solid weaning foods principally facilitates the change and diverse proliferation of intestinal flora, until a stable adult-like colony is attained, around 3 years of age for humans (Guinane and Cotter, 2013; Cryan et al., 2019). Notably, it remains developmentally important for enterocytes to regulate macromolecular entry and strengthen immune defense mechanisms against normal gut flora (Juhl, 2017).

### Barrier to Entry of Noxious Agents From the GIT Following Preterm Birth

Proper development of GIT permeability to macromolecules is important; particularly following preterm delivery. Gut maturation includes the transition from a porous, permeable epithelium, to one that is less endocytic and more resistant to the entry of noxious agents. Normally, the neonatal period, prior to this transition, is characterized by a timed absorptive capacity that allows the unspecific uptake of macromolecules in colostrum, permitting the development of a passive immunity (Sangild, 2001). Preterm offspring face significant challenges including the exposure and vulnerability to diseases, as a result of their underdeveloped gut and highly endocytic enterocytes (Sangild et al., 2013). Stimuli of precocious maturation may be beneficial in the arrest of prolonged penetration of noxious moieties (antigen overload), typically following the use of feeding formulas.

### Prevention of Neonatal Necrotizing Enterocolitis (NEC)

The fetal gut exhibits less metabolic activity compared to the neonatal gut (Neal-Kluever et al., 2019); due to the fetal paucity of luminal contents and a lower oxygen supply compared to that of neonates. Following the onset of lung respiration and circulatory changes, the improved oxygenation in neonatal life supports the development of the gut (Sangild, 2001). However, impaired oxygenation associated with lung immaturity in preterm infants triggers prolonged gut immaturity resulting in gut malfunction and neonatal NEC. Since no cure exists, NEC is a devastating disease (Patel and Denning, 2015). There is need to investigate whether initiating precocious gut maturation may prevent the susceptibility of preterm infants to NEC, and may perhaps address the much-debated use of probiotics for preterm infants (Szajewska and van Goudoever, 2014; Wang et al., 2019).

### Prevention of Weaning Stress

Early weaning of farm animals is an economical bid to manage feed resources, control competition in large litters, maintain dam energy reserves prior to mating and/or improve fecundity (Greenwood and Dunshea, 2009; Kenyon and Cranston, 2017). However, early weaning imposes a detrimental gut challenge and stress on the animal. Early weaning stress, impairs intestinal paracellular barrier function and active absorption in pigs (Wijtten et al., 2011). It also elevates cortisol levels and oxidative stress, decreases immune responses, triggers behavioral deficits, reduces survival, reduces weight gain and increases susceptibility to diseases and parasites, due to insufficient gut maturation in lambs (Napolitano et al., 2008; Yin et al., 2014). Phyto-medicinally induced precocious gut maturation has been reported to improve intestinal function of weaned animals (Prykhod’ko et al., 2009).

### Prevention of Post-weaning Diarrhea

Post weaning diarrhea (PWD) commonly occurs in pigs during the first 2 weeks post-weaning. It presents a significant economic burden for pig farmers due to sudden death, intestinal mucosal damage and reduced growth triggered by enteric invasion and proliferation of various serotypes of enterotoxigenic *Escherichia coli* (ETEC) (Heo et al., 2013). This bacterium attaches to glycoprotein receptors of enteric villi, and releases toxins which increase electrolyte secretion and reduces liquid absorption. The bacterial adhesions and invasions are promoted by the absence of active immunity and gut integrity. The serotypes that are responsible for PWD in piglets also cause diarrheal disease in calves and humans (Heo et al., 2013; Celi et al., 2017). Precociously induced gut maturation may potentially serve as an effective alternative to in-feed antibiotics at reducing the incidence and severity of PWD.

## Developmental Programming and Possible Complications of Precocious Maturation of the GIT Through Medicinal Plants and Phytochemicals

The developmental origins of health and disease (DOHaD) theory postulates that the environment experienced in early life can have lasting effects on the physiology and health of individuals (Heindel and Vandenberg, 2015). Here, environmental factors such as diet during periods of developmental plasticity can trigger alterations in organ and system functionality and if persistent, programming results (Vickers, 2014). The use of medicinal herbs and phytochemicals during critical periods of plasticity may cause epigenetic modifications. For instance, soya products rich in genistein are epigenome modifiers with both time and sex dependent effects (Shankar et al., 2016). In mice, maternal consumption of genistein triggers hypermethylation in the fetal genome, decreasing the prevalence of obesity (Dolinoy et al., 2006). Postnatal genistein exposure, however, regulates methylation and activation of adipogenic genes in female rats increasing susceptibility to obesity (Strakovsky et al., 2014).

Medicinal herbs are often consumed as part of the diet pre-conception and during pregnancy are used as self-medication to alleviate nausea, vomiting, heartburn and constipation, to trigger parturition and to prevent or manage endemic diseases like malaria (Kamatenesi-Mugisha and Oryem-Origa, 2007; John and Shantakumari, 2015; Nergard et al., 2015). Some medicinal herbs including fenugreek, garlic, ginger, chamomile and thistle are used as galactogogues during lactation (Sim et al., 2013; Sevrin et al., 2020). Consumption of medicinal herbs and phytochemicals during these developmental stages leads to their passage from mother to child via the placenta and breast milk (Tsopmo, 2018) and can impact programming and GIT maturation (Patti, 2013; Hanafi et al., 2016). However, intake of these bioactive compounds is often shrouded in secrecy leading to a paucity of data on the potential complications regarding programming and GIT maturation (El Hajj and Holst, 2020).

In humans, increases or decreases in nutrient availability *in utero* and/or during the first 1,000 days after birth increase the susceptibility to metabolic diseases later in life (Marciniak et al., 2017). Phytochemicals and medicinal herbs can increase or decrease nutrient availability and possibly contribute to the development and progression of diseases in adulthood. Soya-containing instant formulas, which contain up to 14 mg of genistein equivalents/liter and are fed to infants with milk allergies (American Academy of Pediatrics, 1998) when fed to piglets for 10 days, decreased enterocyte growth and migration (Chen et al., 2005). Additionally, consumption of ginger, capsaicin, fenugreek, curcumin and mustard by rats lead to increased enzymatic activity of disaccharidases, chymotrypsin and pancreatic lipase (Platel and Srinivasan, 1996, 2000). Maternal supplementation with seaweed-derived polysaccharide (SDP) to sows from the 83rd day of gestation to postnatal day 28, resulted in accelerated growth of the intestine in the piglets (Heim et al., 2015).

Although not expressly investigated in these studies, the reported effects may lead to a decrease or increase in nutrient availability, respectively, which may have potential adverse health effects as reported for similar variations in “normal” nutrients (Agarwal et al., 2018; Baker and Friedman, 2018; Cao et al., 2020).

Studies have highlighted some complications following enteral exposure to PHA (Linderoth et al., 2006; Sureda et al., 2018). Within the first 24 h, oral exposure to PHA disrupted the gut and its mucosa, caused diarrhea and impeded intestinal assimilation of nutrients. However, these early effects are transient and are subsequently accompanied by increased growth and induced maturation of the GIT and pancreas. These undesired effects are a consequence of the PHA binding to the enterocytes and these symptoms disappear within 24–48 h period when the binding has subsided or vanished (Linderoth et al., 2006; Sureda et al., 2018). In contrast, maternal supplementation with SDP reduced diarrhea in piglet during lactation (Heim et al., 2015).

Furthermore, the relationship between gut microbiota and phytochemicals is bidirectional; phytochemicals alter the composition of gut microbiota and they in turn digest normally indigestible molecules into bioavailable molecules, increasing nutrient availability (An et al., 2019; Dingeo et al., 2020). This suggests that phytochemical-induced precocious GIT maturation, which promotes nutrient absorption and availability, may predispose the offspring to metabolic diseases later in life. Therefore, this warrants further investigation as it presents a potential complication of precocious GIT maturation.

Another potentially harmful effect of the phytochemical-gut microbiota interaction is toxification, in which previously non-toxic molecules are converted to toxic products (An et al., 2019). The phytochemicals geniposide, daidzin, and puerarin have been implicated in toxification reactions in various cell lines and animal studies (Kim et al., 1998; Kang et al., 2012). This is an area requiring further exploration.

## Paneth Cells as Phytomedicinal Intermediaries in Gut Maturation

Indeed, the influence of the gut microbiota-axis is pivotal to gut maturation. Intestinal Paneth cells are central means to this effect. They respond to neuronal stimulation, modulate microbiota and are impacted by the flora, oppose the NEC phenotype, sustain a healthy restitutive stem cell niche and develop in par with the gut (Lueschow and McElroy, 2020). Paneth cell number has been correlated to diet such that the Paneth cell numbers in the small intestine decrease following excessive intake of diets laden with fat and carbohydrates (Becerril et al., 2005). With a turnover time of about 15 days (Karam, 1999), these cells are a potential target for orally consumed medicinal plants and phytochemicals which can alter their function and developmental trajectory.

The impact of microbes on Paneth cells has been well documented (Vaishnava et al., 2008). Probiotics, specifically *Lactobacillus casei* CRL 431 (Lc 431) and *L. paracasei* CNCM I-1518 (Lp 1518), administered to mice, increased the activity and number of Paneth cells in the small intestine, and conferred an increased antimicrobial activity against pathogenic bacteria in the gut (Cazorla et al., 2018). Medicinal plants contain phytochemicals which can alter the gut microbiome (Cheung et al., 2020), which influences Paneth cell development. Despite the importance of Paneth cells, and the widespread use of medicinal plants and herbs which could potentially impact the cells, a review of the literature indicates that there is a dearth of direct studies in this respect.

Polyphenols in plants confer health benefits to the metabolic health of individuals and indeed in obese mice, the phytochemical rutin (quercetin) and the prebiotic inulin, were shown to reduce endoplasmic stress in Paneth cells (Guo et al., 2018). Astaxanthin a carotenoid, isolated from crab, shrimp and algae, when administered to immunodeficient mice at different ages from weaning, maintained Paneth cell activity and number within normal ranges (Zhang et al., 2020). Soyabeans and lentils are widely consumed and are a source of leucine which stimulates Paneth cells to secrete α-defensins (Takakuwa et al., 2019) which can impact the gut microbiome. Furthermore, radiation therapy negatively impacts rapidly dividing cells such as those of the GIT, and Paneth cells are not spared (Bala et al., 2015). The leaf extracts from the plant *Hippophae rhamnoides* L. (Sea Buckthorn) were shown to improve Paneth cell survival and function in gamma irradiated rats (Bala et al., 2015).

The potential benefits of medicinal plants and phytochemicals, should be weighed against their potential toxic effects on Paneth cells. *Solanum glaucophyllum* (waxy-leaf night shade) produces active forms of vitamin D3 (Napoli et al., 1977). Dried leaves and extracts from the plant are commonly marketed as a natural source of vitamin-D3. However, it has been shown to be toxic to Paneth cells in rabbits (Zanuzzi et al., 2008). The implications in humans need to be studied further.

### Conclusion

Although this review has generally focused on the potential positive outcomes of precocious maturation of the GIT, there are several studies on the negative impact of phytochemicals and medicinal plants on GIT maturation and subsequent animal health. Nonetheless, the roles some medicinal plants and phytochemicals play in promoting smooth and early maturation of the gut cannot be overemphasized. The knowledge of these stimulatory agents of early maturation is central to understanding the processes featuring gut maturation in order to reap economic benefits, address problems arising from immature guts in farm animals and preterm new-borns that are weaned precipitously.

## Author Contributions

All authors listed have made a substantial, direct and intellectual contribution to the work, and approved it for publication.

## Conflict of Interest

The authors declare that the research was conducted in the absence of any commercial or financial relationships that could be construed as a potential conflict of interest.

## Publisher’s Note

All claims expressed in this article are solely those of the authors and do not necessarily represent those of their affiliated organizations, or those of the publisher, the editors and the reviewers. Any product that may be evaluated in this article, or claim that may be made by its manufacturer, is not guaranteed or endorsed by the publisher.

## Supplementary Material

Supplementary Table 1 Studies investigating the role of medicinal plants and phytochemicals on precocious gastrointestinal tract maturation.
