# Supplementary material for: Medicinal Plants, Phytochemicals, and Their Impacts on the Maturation of the Gastrointestinal Tract
Source: Front Physiol. 2021 Jul 30;12:684464. doi: 10.3389/fphys.2021.684464 (PMC8363294; doi:10.3389/fphys.2021.684464)
Supplement: Supplementary Table 1 — Studies investigating the role of medicinal plants and phytochemicals on precocious gastrointestinal tract maturation. [file Table_1.docx]

**Supplementary data Table 1: Studies investigating the role of medicinal plants and phytochemicalson precocious gastrointestinal tract maturation**

| **Medicinal plants and phytochemicals** | **Animal model** | **Dose** | **Age at intervention** | **Duration of intervention** | **Age at evaluation of parameters** | **Main findings** | **Reference** |
| --- | --- | --- | --- | --- | --- | --- | --- |
| *Hibiscus sabdariffa* | Sprague-Dawley rats | 50, 500 mg/kg; orally | 4 days | 9 days | 14 days | Increased size of small intestine and cecum | Ibrahim et al. (2017) |
| *Ficus thonningii* | Sprague-Dawley rats | 50 mg/kg and 500 mg/kg each of aqueous and methanolic extract; orally | 6 days | 7 days | 14 days | Increased mass of stomach in high dose of methanolic extract, histology revealed dose- dependent increased size of caecal mucosal layers in aqueous and methanolic extract | Dangarembizi et al. (2014) |
| *Aloe vera* | Sprague-Dawley rats | 50 mg/kg and 500 mg/kg each of aqueous and ethanolic extract; orally | 6 days | 8 days | 15 days | Significantly increased cecum size at high dose of aqueous and ethanolic extract, histological examination showed increased thickness of muscularis, submucosa and mucosa layers of cecum, no effect on the height and crypt depth of small intestine | Beyaa et al. (2012) |
| *African potato (Hypoxishemerocallidea)* | Sprague-Dawley rats | 50 mg/kg and 500 mg/kg each of aqueous and ethanolic extract; oral route | 10 days | 5 days | 16 days | High dose of ethanolic extract increased the caecal weight, low dose of the alcoholic extract reduced pancreatic weight | Erlwanger and Cooper (2008) |
| Phytohaemagglutinin (PHA) | Sprague- Dawley rats | 2, 10, 50, 250 µg/g  orally | 14 days | once | Parameters were evaluated after 12h and 72h | High doses of 50 and 250 µg/g shortened SI villi and increased crypt depth after 24h. Observations after 72h include  increased SI length and weight, increased crypt depth. Histology revealed acquisition of non-vacuolated adult-like enterocytes in the distal SI, increased maltase and sucrase activities | Prykhod'ko et al. (2009) |
| Phytohaemagglutinin (PHA) | Sprague- Dawley rats | 0.1 mg/g  orally | 14 days | once | At different time points of 1, 4, 8, and 24 h after intervention | Increased weight of the proximal SI after 24h, 20% decreased spleen weight after 4h and 24h of intervention, decreased pancreatic enzymes after 24h, increased trypsin activity in distal SI, attainment of adult-type FcRn-negative characteristics indicated by a marked decrease in permeability markers (BIgG, HSA and FD4). | Sureda et al. (2018) |
| Phytohaemagglutinin (PHA) | Sprague- Dawley rats | 50 µg/g in 0.9% NaCl orally | 14 days | 3 days | 18 days | Early maturation of sodium transporter, increased proteins in brush border membranes | Kruszewska et al. (2003) |
| Phytohaemagglutinin (PHA) | Sprague-Dawley Rats | 0.05 mg/g in 0.9% NaCl orally | 10, 14 days | 3 days | 13, 17 | Increased weight of SI, increased number of crypts, reduced number of vacuolated enterocytes, decreased macromolecular absorption, acquisition of adult-like intestinal disaccharidase pattern, accelerated growth of pancreas, increased pancreatic protein and trypsin content | Linderoth et al. (2005) |
| Phytohaemagglutinin (PHA) | Sprague- Dawley rats | 0.05 mg/g in 0.9% NaCl orally | 14 days | Single dose | 1-72h after PHA treatment | 1-24h: PHA is bound to the lining of the gut mucosa, altering gut morphology. Reduced disaccharidase activities and macromolecular absorption  1-3d: Reduced binding of PHA, increased uptake by enterocyte. Increased crypt depth, increased disaccharidase activity and decreased macromolecular absorption capacity, Increased pancreatic growth with attendant increase in pancreatic enzymes | Linderoth et al. (2006) |
| Allicin | Pigs (Large Polish White breed) | 1 mg/kg orally | 2 days | 6 days | 8 days | Increased villi height in the duodenum and jejunum, increased surface area of the villus, increased villus number per cm in the distal part of the small intestine, enhanced non-specific defence mechanism, improved terminal body weight. | Tatara et al. (2008) |
| Seaweed-derived polysaccharide | Crossbred pigs (Large White x Landrace genetic lines; Hermitage) | 10 g/day | Maternal supplementation (day 83 of gestation) | 51 days (day 83 of gestation to weaning at day 28) | 28 days (piglets) | Increased villus height in the jejunum and ileum, augmented immune response of suckling piglets, decreased diarrhoeal score | Heim et al. (2015) |

**References**

Beyaa, W., Davidson, B., and Erlwangera, K. (2012). The effects of crude aqueous and alcohol extracts of Aloe vera on growth and abdominal viscera of suckling rats. *Afr. J. Tradit. Complement. Altern. Med.* 9, 553-560. <www.doi.org/10.4314/ajtcam.v9i4.13>

Dangarembizi, R., Erlwanger, K. H., and Chivandi, E. (2014). Effects of Ficus thonningii extracts on the gastrointestinal tract and clinical biochemistry of suckling rats. *Afr. J. Tradit. Complement. Altern. Med.* 11, 285-291. <www.doi.org/10.4314/ajtcam.v11i2.10>

Erlwanger, K. H., and Cooper, R. G. (2008). The effects of orally administered crude alcohol and aqueous extracts of African potato (Hypoxis hemerocallidea) corm on the morphometry of viscera of suckling rats. *Food Chem. Toxicol.* 46, 136-139. <www.doi.org/10.1016/j.fct.2007.07.007>

Heim, G., O'Doherty, J. V., O'Shea, C. J., Doyle, D. N., Egan, A. M., Thornton, K., et al. (2015). Maternal supplementation of seaweed-derived polysaccharides improves intestinal health and immune status of suckling piglets. *J Nutr Sci.* 4, e27. <www.doi.org/10.1017/jns.2015.16>

Ibrahim, K. G., Chivandi, E., Mojiminiyi, F. B., and Erlwanger, K. H. (2017). Aqueous Calyx Extract of Hibiscus sabdariffa: Impact on Growth, Gastrointestinal Morphometry, Liver and Clinical Chemistry of Suckling Rats. *Asian J. Anim. Vet. Adv.* 12, 311-318. <www.doi.org/10.3923/ajava.2017.311.318>

Kruszewska, D., Kiela, P., Ljungh, A., Erlwanger, K. H., Weström, B. R., Linderoth, A., et al. (2003). Enteral crude red kidney bean (Phaseolus vulgaris) lectin--phytohemagglutinin--induces maturational changes in the enterocyte membrane proteins of suckling rats. *Biol. Neonate.* 84, 152-158. <www.doi.org/10.1159/000071950>

Linderoth, A., Biernat, M., Prykhodko, O., Kornilovska, I., Pusztai, A., Pierzynowski, S. G., et al. (2005). Induced growth and maturation of the gastrointestinal tract after Phaseolus vulgaris lectin exposure in suckling rats. *Journal of pediatric gastroenterology and nutrition.* 41, 195-203. <www.doi.org/10.1097/01.mpg.0000172262.05297.98>

Linderoth, A., Prykhod'ko, O., Ahrén, B., Fåk, F., Pierzynowski, S. G., and Weström, B. R. (2006). Binding and the effect of the red kidney bean lectin, phytohaemagglutinin, in the gastrointestinal tract of suckling rats. *The British journal of nutrition.* 95, 105-115. <www.doi.org/10.1079/bjn20051612>

Prykhod'ko, O., Fed'kiv, O., Linderoth, A., Pierzynowski, S. G., and Weström, B. R. (2009). Precocious gut maturation and immune cell expansion by single dose feeding the lectin phytohaemagglutinin to suckling rats. *The British journal of nutrition.* 101, 735-742. <www.doi.org/10.1017/s0007114508035940>

Sureda, E. A., Prykhodko, O., and Weström, B. (2018). Early effects on the intestinal barrier and pancreatic function after enteral stimulation with protease or kidney bean lectin in neonatal rats. *Br. J. Nutr.* 119, 992-1002. <www.doi.org/10.1017/S0007114518000168>

Tatara, M. R., Sliwa, E., Dudek, K., Gawron, A., Piersiak, T., Dobrowolski, P., et al. (2008). Aged garlic extract and allicin improve performance and gastrointestinal tract development of piglets reared in artificial sow. *Ann. Agric. Environ. Med.* 15, 63-69.
